# Community Orientation Scale among Community Health Nurses in Fiji: Scale development and psychometric evaluation

**DOI:** 10.1002/nop2.508

**Published:** 2020-05-25

**Authors:** Sachiko Tanabe, Satoko Yanagisawa, Silina Waqa Ledua, Mereani Tukana

**Affiliations:** ^1^ School of Nursing Kitasato University Kanagawa Japan; ^2^ Graduate School of Nursing & Health Aichi Prefectural University Nagakute Japan; ^3^ Fiji Nursing Council Suva Fiji; ^4^ Former Ministry of Health and Medical Services Suva Fiji

**Keywords:** community nursing, evidence‐based practice, health needs instrument, health promotion, scale development

## Abstract

**Aim:**

To develop and evaluate the reliability and validity of the COSCHN, a scale that aims to measure community orientation among community health nurses in Fiji.

**Design:**

Descriptive cross‐sectional design.

**Methods:**

A self‐administered questionnaire that included the 51 items in the preliminary COSCHN was distributed to community health nurses in Fiji from April–July 2016.

**Results:**

Exploratory factor analysis of 226 responses (77.4% response rate) to the COSCHN revealed 30 items loading on four factors: Community Initiative Promotion, Consensus Building for Community Needs and Activity Goal, Commitment towards Work and Community Members and Mutually Trusting Relationships with Community Members towards Empowerment. Confirmatory factor analysis with high‐order factor modelling revealed a reasonable fit to the data. Cronbach's α values for the COSCHN and the four factors ranged from 0.78–0.94. Weak correlations were noted for concurrent validity, while known‐groups validity and time stability were generally satisfactory.

## 1 INTRODUCTION

Fiji currently faces a triple burden of health issues: communicable diseases, non‐communicable diseases (NCD) and injuries; this is a common trend observed in a growing number of low‐ and middle‐income countries (Wiseman et al., 2017). To provide communities with a wide range of services to accommodate the varying health needs of each, the Ministry of Health and Medical Service (MOHMS) in Fiji applied the key framework of the Healthy Island Policy (World Health Organization Regional Office for the Western Pacific [WPRO], 2015) to develop a national strategy to form the basis for reorienting primary healthcare (PHC) delivery to people in communities through expanded partnerships between health professionals and local communities (MOHMS, 2015). As front‐line PHC providers for the MOHMS, community health nurses (CHNs) in Fiji are responsible for providing the necessary health services to those in the communities.

Understanding the determinants of health, and the nature and extent of the community needs, is a fundamental demand of CHNs, who are required to make sound decisions about health (Institute of Medicine Committee for the Study of the Future of Public, 1988). Community assessment is a core nursing process that requires a logical and systematic approach to identify community health needs, strengths and resources for community health services (Shuster, 2010). Various models for management of community health needs have been developed and used to enable effective community assessment and its use for community health services (Green & Kreuter, 2005; Kreuter, 1992). One of the models aims specifically to achieve community orientation (CO), defined as the organization‐wide generation, dissemination and response to community intelligence to address present and future community health needs (Proenca, 1998). The concept is derived from market orientation, which assumes that the best way to understand and satisfy consumer needs is to develop a closeness to the market (Kohli & Jaworski, 1990). Proenca (1998) noted that CO must be carried out in collaboration with other community systems and organizations, since community health needs are both affected by this and beyond the control of the healthcare system. Successful collaboration requires comprehensive competency that should fit a nation's healthcare system and culture and the responsibilities of CHNs to deliver these services (Lin, Hsu, Li, Mathers, & Huang, 2010). Although a measurement tool to assess CO was developed and widely in use for health service organizations in the United States (Ginn, Shen, & Moseley, 2009), no research has been published about CO among CHNs in Fiji.

Several factors affect CHN task performance. With the recent health policy reform, clinical services have been decentralized to health centres (MOHMS, 2015). This shift has required CHNs to spend more time providing outpatient care at their facilities. The working environment can impede their work in that CHNs usually work alone in their assigned communities, with few opportunities to mimic and receive timely advice from senior CHNs, except during special events such as outreach team visits. Senior CHNs have developed their own competency through meaningful yet challenging experiences that have sharpened the effective management of community health activities. However, these experiences are intangible and thus difficult to convey verbally to junior CHNs, especially given the working conditions of CHNs in Fiji. Therefore, identifying detailed characteristics of CO for CHNs in Fiji could help them to gauge how much attention must be paid to understand community health needs and manage community activities.

## 2 BACKGROUND

To identify detailed characteristics and develop a framework for CO among CHNs in Fiji, we conducted semi‐structured individual interviews with 20 participants, nine CHN supervisors/managers/lecturers at a nursing school, three novice CHNs, three policymakers in the MOHMS and three community members (Tanabe, Yanagisawa, Waqa‐Ledua, & Tukana, 2019). Data were analysed using descriptive qualitative methods. Each occupation comprised a unit for analysis. As most codes were extracted from expert CHNs, this group was set as the main group for analysis. None of the codes from expert CHNs contradicted those of other groups. Extracted sentences were merged into 57 final codes, which were then consolidated to form 12 subcategories. Finally, these were combined into three categories: Trusting Relationships, Commitment and Activity Management. Trusting Relationships and Commitment were inter‐related and serve as a foundation to promote Activity Management. Reflection and a sense of self‐accomplishment during Activity Management further strengthened Commitment and Trusting Relationships. Therefore, each of the three main characteristics is linked to others and circulated as experienced.

Competency is an underlying characteristic of an individual that is causally related to criterion‐referenced effective and/or superior performance in a job (Spencer & Spencer, 1993). When evaluating competency, it is not the performance and action that are important, but rather why and how one performs (Streiner, Norman, & Cairney, 2015). Collecting reasons and thoughts underlying the actions and identifying a thought pattern can maximize the generalizability to various outcomes of actions rather than simply listing superficial actions (McClelland, 1973). Thus, development of a self‐assessment scale specific to CHNs in Fiji to measure thought patterns such as one's attitude, value and self‐image towards CO could allow CHNs to understand these features within themselves as they relate to community health activities, allowing them to be strengthened and educated. To this end, the purpose of the present study was to develop the Community Orientation Scale among Community Health Nurses (COCHN) and assess its reliability and validity.

The present study was part of a larger research project, entitled “Community Orientation among Community Health Nurses in Fiji: Scale Development and Influencing Factors.” Data for our analysis were extracted from a cross‐sectional survey conducted from April–July 2016 in Fiji.

## 3 DESIGN

This study was a descriptive cross‐sectional design.

## 4 METHODS

### 4.1 Participants

The survey was administered to all CHNs in Fiji. During the study period, the total number of CHNs in Fiji was 269. Although the whole CHN population was included in this study, we assessed the number of participants since the factor pattern that emerges from a large‐sample factor analysis will be more stable than that from a smaller sample (DeVellis, 2016). When determining sample size for factor analysis, one must consider the research questions, type of model, the number of factors or items and more (Mokkink et al., 2018). In general, however, as a checklist for assessing methodological quality of studies of measurement properties has proposed that a ratio of at least 5 participants per item and more than 100 participants would represent an adequate sample size (Prinsen et al., 2018), we followed these guidelines. As described later, the preliminary COSCHN consisted of 51 items; therefore, the necessary sample size was set to 255 participants. Collection rate was not anticipated to be high, due to geographical challenges and limited means of transportation. Therefore, in addition to all 269 active and practising CHNs during the study period, we also administered the survey to 31 former CHNs who had transferred from CHN work to other positions within 1 year prior to the survey administration (hereafter, “former CHNs”). Contact with former CHNs was attempted after obtaining information from practising CHNs when the questionnaires were distributed. In total, the target number of research participants was set to 300 CHNs (269 practising CHNs and 31 former CHNs).

In this study, CHNs in Fiji comprised zone nurses and district nurses who provide both basic clinical treatment and health‐promoting services to community members and settings in their respective environments in the assigned area. Zone nurses work at health centres with other health workers, the latter of which includes CHN colleagues and allied health personnel such as physicians or nurse practitioners, nutritionists and health inspectors. District nurses work alone in nursing stations in areas without easy access to health centres.

### 4.2 Questionnaire components

The self‐administered questionnaire comprised four parts:

1. 51 items of the preliminary COSCHN
2. Sociodemographic background: age, position (zone/district nurse) and educational achievements
3. Items to assess known‐groups validity: self‐reported supervisor's competency assessment measured on a 10‐point scale where 1 is “very bad” and 10 is “excellent.”
4. Items to assess concurrent validity: six items about normative commitment taken from the academic version of the Three Component Model Employee Commitment Survey Normative Commitment Scale (TCM‐NCS, Meyer & Allen, 2004). This measures the sense of obligation to the organization (such that employees with strong normative commitment remain because they feel they ought to do so) along with a 7‐point Likert scale for which 1 is “strongly disagree” and 7 is “strongly agree.” For one item, this is reversed, such that 1 is “strongly agree” and 7 is “strongly disagree.” Evidence for reliability and validity of the scale accumulated through years of research (Allen & Meyer, 1996). The normative commitment scale was chosen for concurrent validity because normative commitment was one of the main components of the CO framework developed in the previous study. Permission to use the TCM‐NCS was obtained from the authors’ organization.

### 4.3 Preliminary Community Orientation Scale among Community Health Nurses in Fiji

The preliminary COSCHN was created based on the conceptualized framework in the previous qualitative descriptive study (Tanabe et al., 2019), as described above. Final codes were used as items for the preliminary COSCHN. Six items were excluded from the 57 final codes, as they contained similar content to other items. In the end, 51 items were created. Four items were reversed meaning of the statements to reduce response bias. The items were assessed along a 7‐point Likert scale to reflect the degree of practice, whereby 1 is “not at all” and 7 is “extremely.” While a higher number reflected a higher level of practice, this was reversed in the case of four reversed items for which a higher number reflected a lower level of practice.

To verify content validity, an expert review and a pilot test were conducted. The questionnaire was checked by eight experts: four medical and nursing managers of the MOHMS, one senior nursing lecturer and three public health researchers. A pilot test was also conducted to examine about its clarity of explanation, response time and questions with a low response rate. The questionnaire was distributed to 29 CHNs selected by convenience sampling; 28 forms were returned. Some text of the 51 items was modified to reflect the comments from the experts and the pilot test participants. The changes in items were made after discussion among co‐researchers.

### 4.4 Data collection

Questionnaires with return envelopes were distributed to each individual CHN by either the chief researcher or nursing managers. The questionnaire forms were collected 1 week after distribution from CHNs who did not complete them immediately. The chief researcher also visited former CHNs when introduced to them by practising CHNs. Orientation and instruction was given at the time of distribution. Forms were distributed and collected by nursing managers when the chief researchers were not available. Nursing managers were instructed that they were to neither insist on submission nor confirm responses. Participants of the survey received F$6 (approximately US$3.0) phone vouchers. Nursing managers who cooperated with distribution and collection received F$30 (approximately US$15.0) phone vouchers.

The retest survey was conducted 1 month after CHNs returned the questionnaire forms. The target population comprised CHNs in the central division who submitted a consent form for the retest, which followed the same procedure as the survey.

### 4.5 Data analysis

Of the collected questionnaires, those with no responses or multiple answers in the preliminary COSCHN were eliminated. All items with reversed scores (four in the preliminary COSCHN and one in the TCM‐NCS) were analysed after rescoring to reflect the reversed points.

Initially, sociodemographic background was analysed. Subsequently, item analysis was conducted. Skew, ceiling effect and floor effect were analysed to examine score distribution and normality. Skew of each item was determined using the exclusion criteria of either >1.0 or <−1.0. Ceiling effect was calculated by the sum of the mean and standard deviation (*SD*), and any items exceeding the maximum score (7) were excluded. Floor effect was calculated by subtracting *SD* from the mean, and any items scoring below the minimum score (1) were excluded. The item‐total correlation analysis was conducted to eliminate items that did not correlate with a given component by determining Pearson's correlation coefficients (Pearson's *r*) between an individual item and the total score without that item; any items for which Pearson's *r* < .2 were eliminated. Good–poor analysis was conducted to verify the discriminative power of each item by Welch's *t* test and items that showed no statistically significant differences (*p* < .05) were eliminated. Correlations within items were assessed to eliminate items that were too similar by calculating Pearson's *r* (one of the two items was eliminated if Pearson's* r* was >.7 between them).

Subsequently, exploratory factor analysis (EFA) was conducted to assess construct validity with the exclusion point for factor loading set to 0.4. Items with factor loading that was just slightly lower than the exclusion point were reconsidered and carefully examined. Extracted factors were named. Next, confirmatory factor analysis (CFA) was conducted to determine the factor structure. The fitness of models was assessed using the following: *p* value of CMIN chi‐square (*χ* ^2^), goodness‐of‐fit index (GFI), adjusted goodness‐of‐fit index (AGFI) and root mean square error of approximation (RMSEA). Confidence coefficients, Kaiser–Meyer–Olkin (KMO) and Cronbach's α were calculated to measure suitability of items. Known‐groups validity was examined by Welch's *t* test. Concurrent validity was examined by Pearson's r between TCM‐NCS and a factor indicating commitment.

For reliability assessment, internal consistency was assessed using Cronbach's α for COSCHN and factors. Test–retest reliability was computed as a measure of stability reliability. Analyses were performed by SPSS 24.0 for Windows and Amos 24.0.

### 4.6 Ethical considerations

This study was approved by the Fiji National Health Research Ethics Committee (2016.6.NW) and a University Research Ethics Committee (No. 27APU‐SIC6‐32).

## 5 RESULTS

### 5.1 Respondent characteristics

In total, questionnaire forms were distributed to 292 CHNs (268 current and 24 former CHNs) and 250 (from 226 current and 24 former CHNs) were collected (85.6%). Data from 226 respondents (205 current and 21 former CHNs) were considered valid for analysis (77.4%). Descriptive statistics of the study population are presented in Table 1. Of the valid respondents, 142 (62.5%) respondents reported working as zone nurses and 93 (37.7%) as district nurses. Mean age was 31.0 years (SD5.7). Approximately 80% (*N = *177, 78.3%) of total respondents were reportedly ≥ 34 years. Populations covered by respondents ranged in size from <1,000–≥11,000 people. Approximately 70% (156, 68.6%) of respondents were assigned to communities with <5,000 people in the population. Mean experience in the current position (or as a former CHN) was 3.25 years (SD3.3). Mean experience working as a CHN was 4.4 years (*SD* 4.4) and that as any nursing experience (clinical and public health) was 7.8 years (*SD* = 5.2). Most respondents (*N = *225, 99.6%) had obtained an associate degree in nursing, six (2.7%) had obtained a bachelor's degree in nursing science, and four (1.8%) had obtained a midwifery licence.

**TABLE 1:** Descriptive statistics of the study population (*N* = 226)

Items	Characteristics	No.	%
Position			
	Zone nurse	130	57.5
	District nurse	75	33.2
	Former zone nurse	11	4.9
	Former district nurse	10	4.4
	No answer	0	0
Age (Mean ± *SD*) 30.96 ± 5.72			
	<25 years	19	8.4
	25–29 years	81	35.8
	30–34 years	77	34.1
	35–39 years	31	13.7
	40–44 years	10	4.4
	45–49 years	5	2.2
	50–54 years	2	0.9
	No answer	1	0.4
Target population			
	<1,000	40	17.7
	1,000–2,999	69	30.5
	3,000–4,999	46	20.4
	5,000–6,999	28	12.4
	7,000–8,999	24	10.6
	9,000–10,999	8	3.5
	≥11,000	5	2.2
	No answer	6	2.7
Years of experience as a current/former zone/district nurse (Mean ± *SD*) 3.25 ± 3.25			
	<3 years	136	60.2
	3–5 years	55	24.3
	6–8 years	17	7.5
	9–11 years	9	4.0
	≥12 years	3	1.3
	No answer	6	2.7
Total years of experience as a zone/district nurse (Mean ± *SD*) 4.38 ± 4.39			
	<3 years	113	50.0
	3–5 years	59	26.1
	6–8 years	18	8.0
	9–11 years	16	7.1
	≥12 years	12	5.3
	No answer	8	3.5
Total years of experience as a nurse (including clinical and public health) (mean ± *SD*) 7.81 ± 5.18			
	<2 years	37	16.4
	3–5 years	64	28.3
	6–8 years	40	17.7
	9–11 years	41	18.1
	≥12 years	32	14.2
	No answer	12	5.3
Education and Licences			
	Associate's degree/Registered Nurse (RN)	225	99.6
	Bachelor's degree in Nursing Science/RN	6	2.7
	Midwifery	4	1.8
	Other	33	14.6
	No answer	0	

### 5.2 Item analysis

In total, 14 items were discarded from the preliminary COSCHN: seven due to skew, eight due to the ceiling effect and four due to I‐T correlation analysis, yielding a final total of 37 items included in the preliminary COSCHN.

### 5.3 Development of the COSCHN

#### 5.3.1 Exploratory factor analysis

EFA was conducted using the maximum likelihood technique and promax rotation. The eigenvalue rule (DeVellis, 2016) and scree plot of an initial EFA revealed four factors. As a result of the first EFA, seven items were discarded, and as a result of the second factor analysis with 30 items, one item was discarded. Factor loading for the third factor analysis with 29 items exceeded 0.4 for all items. All discarded items were examined before finalizing the item list. In so doing, Item 25 (“I pay attention to vulnerable people and minority groups when collecting and analysing information (hereafter, Vulnerable Sensitivity)”) was reconsidered because of its significance and because the factor loading was barely below 0.4 (0.399) in the first analysis. Therefore, the fourth factor analysis was conducted to include Vulnerable Sensitivity. This analysis revealed a factor loading of 0.397 for Vulnerable Sensitivity, with all other items exceeding 0.4 (Table 2). Vulnerable Sensitivity was tentatively included in the analysis, but was discussed later, in the CFA that followed.

**TABLE 2:** Factor analysis: Community Orientation Scale for Community Health Nurses in Fiji (4 factors, 30 items, total overall Cronbach's *α* = 0.935)

Items	Factors			
	1	2	3	4
Factor 1 Cronbach's *α* = 0.861				
35 I try to participate in community health activities as a participant	0.711			
36 I try to involve other health alliances and organizations to work together for community health activities	0.700			
34 I try to make community health activities attractive to people who are currently not interested in them	0.613			
38 I try to find out how community health activities change community members	0.611			
33 I try to find and train appropriate people to take roles in community health activities	0.590			
39 I seek feedback from participants after community health activities	0.569			
37 I continue to find a way to provide services although currently there are no systems in any organizations to support community members	0.448			
Factor 2 Cronbach's *α* = 0.885				
23 I try to collect information and familiarize myself with other organizations and officers in/for communities		0.974		
21 I collect information about how community members want to spend their lives in the future		0.712		
22 I collect information about people's views and beliefs, and factors that affect their lives		0.703		
15 I try to regularly contact resource people in order to get information and discuss about community health situations		0.576		
17 I try to actively inform issues of concern to resource people		0.506		
24 I try to find connections between social problems and health problems of communities		0.499		
20 I try to ask/check with various sources about issues/health problems in communities		0.485		
31 I try to discuss with resource people and set up goals for mutually understanding the direction of community health activities		0.479		
25 I pay attention to vulnerable and minority groups when collecting and analysing information		0.397		
Factor 3 Cronbach's *α* = 0.817				
43 I try to be a role model for community members			0.704	
46 I share new knowledge and experiences with colleagues			0.659	
50 I am aware of my own strengths and weaknesses			0.583	
41 I try to take immediate action when recognizing issues in my area			0.553	
6 Despite obstacles and limitations, I do my best to fulfil promises that I have made to the community members			0.523	
19 I keep in mind that it takes time for people to change their behaviour towards healthy lifestyles and I need to make continuous efforts to encourage this			0.508	
7 I try to give useful information especially at a first meeting with community members			0.448	
Factor 4 Cronbach's *α* = 0.787				
10 I try to be conscious (sensitive) of people's feelings and emotions and show empathy				0.649
2 I listen to community members rather than talking				0.551
1 I respect community members under any situation				0.537
8 I actively try to contribute to social activities in communities				0.528
4 I review my own attitude to community members every day				0.511
9 When people don't follow my advice, I try to understand their situation				0.499
3 When visiting communities, I try to communicate with as many people as possible in addition to the original purpose such as domiciliary case visits				0.479
Factor correlations	1	2	3	4
1	–	0.625	0.553	0.419
2		–	0.641	0.561
3			–	0.486
4				–

The conceptual framework of CO among CHNs in Fiji as determined by the previous study consisted of three categories: Activity Management, Trusting Relationships and Commitment. However, the EFA divided Activity Management into two factors. As Factor 1 indicated the efforts by CHNs to promote and evaluate ownership among community members during community activities, Factor 1 was named Community Initiative Promotion (hereafter, Initiative Promotion). As all nine items of Factor 2 indicated information collection and planning, Factor 2 was named Consensus Building for Community Needs and Activity Goal (hereafter, Consensus Building). As all items in Factor 3 pertained to responsibilities towards tasks and community members, Factor 3 was named Commitment towards Work and Community Members (hereafter, Commitment). All seven items of Factor 4 (items 1–4, 8–10) belonged to Trusting Relationships in the previous study. Specifically, items 1–4 pertained to familiarizing community members with nurses, items 9 and 10 pertained to showing empathy and trying to understand situations, and item 8 pertained to the presence of a CHN conveying reliability to community members. As such, Factor 4 was named Mutually Trusting Relationships with Community Members towards Empowerment (hereafter, Trusting Relationships).

#### 5.3.2 Confirmatory factor analysis

**FIGURE 1:**
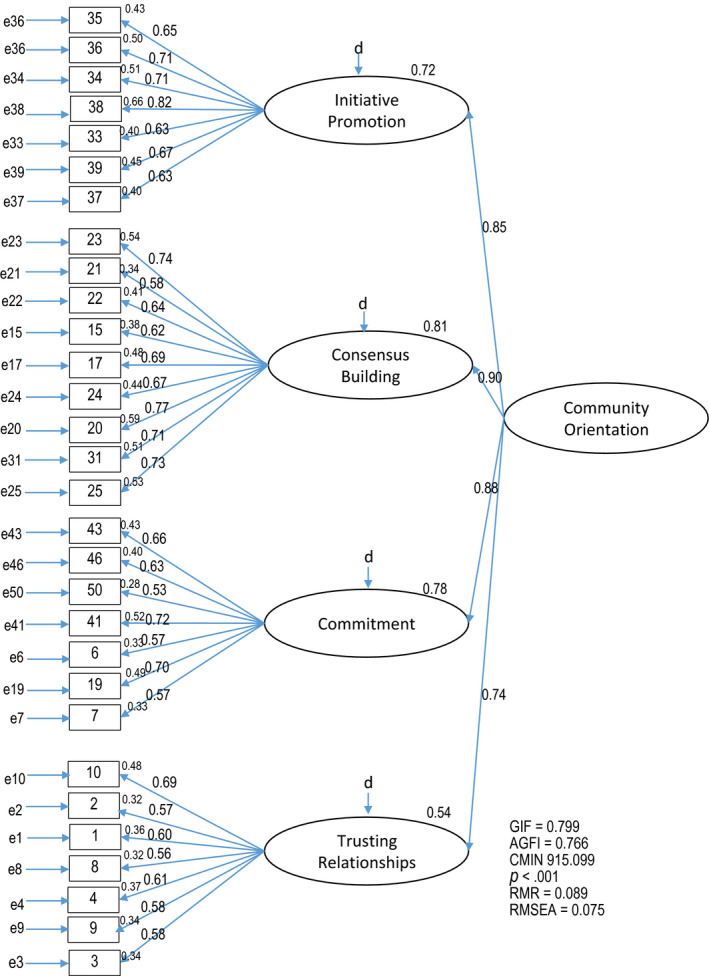
Confirmatory factor analysis: Model 1 (30 items) Community Orientation Scale among Community Health Nurses in Fiji

CFA for the proposed two model of COSCHN was conducted by covariance structure analysis. High‐order factor modelling was set using four factors extracted in the EFA as latent variables and CO as a superordinate (high level) concept. Thirty items were substituted for observation variables (Model 1, Figure 1), and standardized estimates were used in the figures. Path coefficients from the superordinate concept to the four factors ranged from 0.74–0.90, while those from the four factors to the observation variables ranged from 0.63–0.81 in Initiative Promotion, 0.58–0.74 in Consensus Building, 0.53–0.72 in Commitment and 0.56–0.69 in Trusting Relationships. Statistically significant differences were noted for all 30 path coefficients <0.001. Multiple correlation coefficients (coefficients of determination) for factors 1, 2, 3 and 4 were 0.72, 0.81, 0.78 and 0.54, respectively. Of the goodness‐of‐fit indicators, *p* of CMIN was <0.001, GFI was 0.799, AGFI was 0.767 (exceeding the GFI), and RMSEA was 0.075.

**FIGURE 2:**
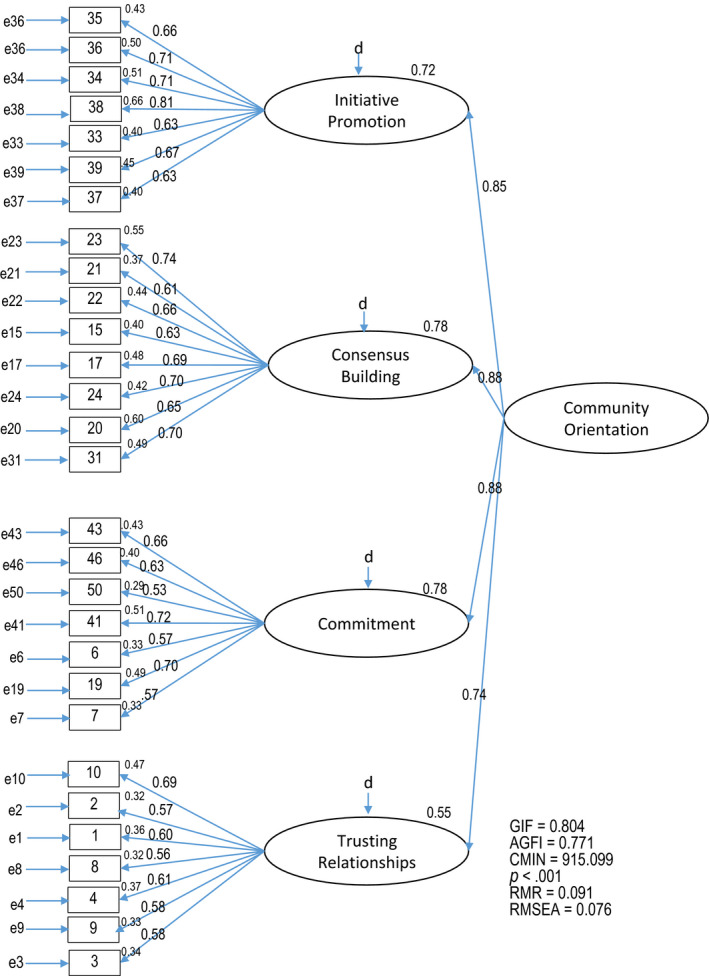
Confirmatory factor analysis: Model 2 (29 items) Community Orientation Scale among Community Health Nurses in Fiji

Further analysis was conducted to confirm the appropriateness of the inclusion of Vulnerable Sensitivity (Item 25). Modified high‐order factor models were proposed with 29 items excluding Vulnerable Sensitivity (Model 2, Figure 2). A comparison of goodness‐of‐fit indicators is presented in Table 3. Model 2 showed the better GFI (>0.8) and AGFI, while Model 1 showed the better RMR and RMSEA; however, the differences were sufficiently small. Therefore, this comparison revealed no remarkable differences between the two models.

**TABLE 3:** Comparison of the two models

Model	GFI	AGFI	*χ* ^2^	RMR	RMSEA	Path coefficients			
						CO → F1	CO → F2	CO → F3	CO → F4
						F1 → item	F2 → item	F3 → item	F4 → item
1	0.799	0.766	*p* < .001**	0.089	0.075	0.85	0.90	0.88	0.74
						0.63 – 0.82	0.58 – 0.74	0.53 – 0.72	0.56 – 0.69
2	0.804	0.771	*p* < .001**	0.091	0.076	0.85	0.88	0.88	0.74
						0.63 – 0.81	0.63 – 0.77	0.53 – 0.72	0.56 – 0.69

Note

Model 1: COSCHN with 30 items.

Model 2: COSCHN with 29 items excluding Item 25 (Vulnerable Sensitivity).

CO → F1: Path coefficient from Community Orientation to Initiative Promotion.

CO → F2: Path coefficient from Community Orientation to Consensus Building.

CO → F3: Path coefficient from Community Orientation to Commitment.

CO → F4: Path coefficient from Community Orientation to Trusting Relationships.

F1 → item: Path coefficient from Initiative Promotion to items.

F2 → item: Path coefficient from Consensus Building to items.

F3 → item: Path coefficient from Commitment to items.

F4 → item: Path coefficient from Trusting Relationship to items.

** *p* < .01.

Confidence coefficients were calculated for Models 1 and 2 (Table 3). Comparison of the KMO sample validity accuracy revealed coefficients of 0.905 for Model 1 and 0.899 for Model 2, yielding a 0.006 higher coefficient for Model 1. Comparison of Cronbach's α values for COSCHN revealed *α* values of 0.94 for Model 1 and 0.932 for Model 2, yielding a 0.003 difference between models. Comparison of Cronbach's *α* for the models when they included Vulnerable Sensitivity revealed *α* values of 0.89 for Model 1 and 0.873 for Model 2, revealing a 0.012 difference between models. Cumulative contribution rates were 53.34% for Model 1 and 53.37% for Model 2, yielding a 0.03% difference. As none of the comparisons showed much change, we concluded that Model 1 that includes Vulnerable Sensitivity should be used for COSCHN (Table 4).

**TABLE 4:** Comparison of reliability for Models 1 and 2

	Model 1 (30 items)	Model 2 (29 items)
KMO sample validity accuracy	0.905	0.899
Cumulative contribution (%)	53.335	53.369
Cronbach's α		
Whole scale	0.935	0.932
Consensus Building (Factor 2)	0.885	0.873

Note

KMO, Kaiser–Meyer–Olkin.

#### 5.3.3 Known‐group validity assessment

To test the known‐groups validity, Welch's *t* test was conducted. Self‐reported supervisor assessments were divided by a median value (8). Significantly higher scores for COSCHN (*p* = .010) were observed in the group with higher scores for self‐reported supervisor competency assessment (Table 5).

**TABLE 5:** Known‐groups validity by supervisor competency assessment

Items	Score	Number	Mean ± *SD*	*p* value
COSCHN	1–7	87	153.33 ± 21.18	.010*
	8–10	108	161.38 ± 21.50	

* *p* < .05 (two‐tailed).

#### 5.3.4 Concurrent validity assessment

To test concurrent validity, Pearson's *r* values were calculated for TCM‐NCS and Commitment. Of the 226 valid respondents, data from 10 were discarded due to missing values in the TCM‐NCS; data from the remaining 219 were used in the analysis. The correlation coefficient was *r* = .263 (*p* < .001) between TCM‐NCS and Commitment. Given the possibility that the reversed item in the TCM‐NCS might be misinterpreted, additional calculations were made using five items of the TCM‐NCS after discarding the one reversed item. This yielded a slightly higher correlation coefficient of *r* = 0.284 (*p* < .001).

#### 5.3.5 Reliability assessment

To test for internal consistency, Cronbach's *α* values were calculated (Table 2). Cronbach's *α* for COSCHN and factors 1, 2, 3 and 4 were 0.935, 0.861, 0.885, 0.817 and 0.787, respectively. No increase was noted in the Cronbach's *α* for any item when it was removed. Correlation coefficients ranged between 0.419–0.641.

To analyse time stability, a retest was conducted among 74 participants who submitted retest consent forms. Of the 74, 54 returned questionnaires (73.0% response rate), 46 of which were valid forms (62.2% valid response rate). Mean COSCHN for the retest was significantly higher (*p < *.001) than that of the original test, with a Pearson's r of 0.519.

## 6 DISCUSSION

In this study, we developed the COSCHN and examined its reliability and validity. This scale measures CO among CHNs in Fiji, with the aim to promote effective management of community health activities.

The EFA identified a four‐factor model from the COSCHN (Figure 1), even though the conceptual framework of CO among CHNs in Fiji in the previous study consists of three main characteristics. This discrepancy is primarily due to the division of Activity Management in the previous study into two factors: (a) information collection and planning and (b) preparation, implementation and evaluation. This division is also in line with the two distinctive capabilities needed to achieve a higher degree of CO as proposed by Proenca (1998): community sensing and community linking. Community sensing is the ability to learn more about community through the structured, ongoing process of tracking community events and trends and is necessary in the information collection process. Community linking indicates the ability to create and manage close relationships with communities. This is particularly important when preparing and implementing activities together with community members and collect honest feedback from them. Factor validity was thereby confirmed. Next, COSCHN was assessed by CFA with high‐order modelling. CMIN chi‐squared test of the path diagram of COSCHN yielded a *p* < .001, revealing that the data were not suitably fitted to the model. However, multiple correlation coefficients of the four factors ranged from 0.81–0.54 in Model 1, which was deemed acceptable. Statistically significant differences were observed for all 30 path coefficients at a level of 0.1%, and Model 1 showed a higher AGFI (0.766) than GFI (0.799) with both values at nearly 0.8. The RMSEA of 0.075 was also interpreted to represent a reasonable fit. Considering the EFA and CFA, the structure of COSCHN demonstrates a reasonable fit with the conceptual framework, confirming the construct validity.

Our analysis concluded that Vulnerable Sensitivity was included in the COSCHN. Several studies pointed out persistent issues with gender inequality, poverty and ethnicity in Fiji (Chattier, 2005; Wiseman et al., 2017). The MOHMS highlights equity as a main value (MOHMS, 2015). The WHO recommends health professionals in Fiji to increase awareness of issues pertaining to gender norms and inequality in disease perpetuation (MOHMS, 2015; WPRO, 2011). Therefore, CHNs are unable to improve health situations without exercising principles of fairness to all population; it is essential that CHNs examine health situations and environments to identify disparities among community members.

Known‐groups validity was tested by self‐reporting supervisor competency assessments. As CHNs with higher supervisor assessment had significantly higher COSCHN scores than those with lower supervisor assessment, known‐groups validity was confirmed to be reasonable.

Concurrent validity as evaluated by TCM‐NCS Pearson's *r* for the TCM‐NCS and Commitment in COSCHN showed weak correlations with statistically significant relationships (*r* = 0.263, *p* < .01). This could be explained by the fact that the TCM‐NCS was developed in the United States and such perceptions and wording pertaining to commitment may vary from those of Fiji. Another reason could be that the TCM‐NCS measures commitment to organizations, while Commitment in the COSCHN revealed that CHNs perceived obligations as those not only to their organizations, or the MOHMS, but to their profession as a nurse and to community members. As such, TCM‐NCS and Commitment in the COSCHN did not measure the exact same parameters, yielding weak correlations.

Internal consistency was evaluated using Cronbach's *α* values. Our analysis revealed *α* values of 0.94 for COSCHN and that ranging between 0.79–0.89 for the four factors. A Cronbach's *α* exceeding 0.7 indicates high reliability (Oshio, 2004). Therefore, the internal reliability was confirmed.

Time stability was tested among 46 patients. The necessary sample size was found to be 29 for a correlation coefficient of 0.5, an α level of 0.05 (two‐tailed) and a β level of 0.2 (Browner, Newman, & Hulley, 2007). Thus, the analysis was confirmed to have been conducted with a sufficiently large sample population. Score of the retest was significantly higher than that of the original test (*p < *.001). There were several seminars and training workshops for CHNs during the test period that could affect response of the retest, however the correlation coefficient of 0.59 that interpreted moderate correlation with the original test and the retest.

## 7 IMPLICATIONS FOR PRACTICE

The COSCHN can serve as a self‐assessment tool for CHNs to understand and review own thought pattern towards collection, dissemination and response to community health needs. Such assessment encourages CHNs to reflect on and identify potential areas requiring growth with regard to manage community health needs. It also enables supervisors to understand CHNs not only through their observations but also from the CHN’s point of view as rated items in the COSCHN. This more comprehensive understanding of CHNs will help supervisors to provide reasonable advice and practical support how to improve community health activities.

In addition to the use of the COSCHN, we would recommend increasing the number of presentation opportunities pertaining to CO‐based community health activities among CHNs. Supervisors should be proactive in providing discussion opportunities in their regular CHN meetings. Annual or biannual nursing forums within divisions and the nation are also recommended. Such presentations would be culturally and environmentally adaptable to the audiences, allowing CHNs to upgrade effectively their practical knowledge and skills. Presenters would expand their competencies through their preparation and become more motivated by these opportunities.

The COSCHN can be used as a reference for developing CO scales for other countries as well. It is especially adaptable for Pacific island countries, where the WPRO Healthy Island Policy is applied to health strategic plans for each country and having similar geographical and climatological features. The process of scale development begins with an examination of its framework and the items by a panel of experts. Scale development for the COSCHN should be finalized through a full assessment of the reliability and validity of the revised version.

Finally, we would recommend that the MOHMS takes steps to improve the accessibility of epidemiological information that could promote CO among CHNs in Fiji. As the MOHMS has recently introduced an online information management system, CHNs should be able to access this database for their division and nationwide. Easy access to the system will allow CHNs to obtain the latest statistics for their areas to analyse trends and compare them with those in other areas, allowing them to assess better the community health needs.

## 8 LIMITATIONS

Our analysis included data from 21 former CHNs as valid respondents who had transferred within the year prior to the survey administration. Their current tasks could have influenced their responses, yielding responses that may have differed from those they may have produced as CHNs. However, the questionnaires were distributed to all CHNs except one CHN who were stationed in the outer island, with most (90.71%) comprising active CHNs (Table 1). Therefore, our analysis was fairly reflective of the views of CHNs. Second, all reversed items in the preliminary COSCHN were discarded due to the negative I‐T correlation. The wording used for reversed items may not be suitable for CHNs in Fiji, as it may cause misinterpretation. We surmise that the wording must be adapted to be more culturally appropriate, so that CHNs are better able to comprehend fully the content and think deeply about the meaning and context. Third, concurrent validity was not supported. This could be because TCM‐NCS was to measure different characteristics of commitment from those measured by the COSCHN, as discussed above. Forth, time stability was not fully supported due to seminars and workshops for CHNs during the retest period.

Further investigation and modification are needed to refine the COSCHN to increase its potential for generalizability. Also, the COSCHN is applicable only to zone and district nurses in Fiji. Future studies should examine the application of the COSCHN to other nursing positions in Fiji, and those in other Pacific island countries.

## 9 CONCLUSIONS

The present study developed the COSCHN, scale to measure thought patterns such as one's attitude, value and self‐image towards management of community health activities, and examined its reliability and validity. Construct validity, known‐groups validity and internal consistency were supported. This scale can be used for self‐assessment and discussion between CHNs and their supervisors. Further research is necessary to refine the scale and increase the potential for generalization.

## CONFLICT OF INTEREST

The authors declare no financial or other conflicts of interest.

## AUTHOR CONTRIBUTIONS

ST and SY were responsible for the study conception, design and drafting of the manuscript. ST performed the data collection. All authors performed the data analysis. SY made critical revisions to the manuscript and supervised the study. All authors read and approved the final manuscript.

## References

[nop2508-bib-0001] Allen, N. J. , & Meyer, J. P. (1996). Affective, continuance and normative commitment to the organization: An examination of construct validity. Journal of Vocational Behavior, 49(3), 252–276. 10.1006/jvbe.1996.0043 8980084

[nop2508-bib-0002] Browner, W. S. , Newman, T. B. , & Hulley, S. B. (2007). Estimating Sample Size and Power: Applications and Examples In Hulley S., Cumming S., Browner W., Grady D., & Newman T. (Eds.), Designing clinical research (pp. 65–96). Philadelphia, PA: Lippincott Williams & Wilkins.

[nop2508-bib-0003] Chattier, P. (2005). Understanding poverty from a gender perspective: Thinking 'small' through Paaru's story. Fijian Studies: A Journal of Contemporary Fiji, 3(2), 249–276.

[nop2508-bib-0004] DeVellis, F. R. (2016). Factor analysis In Bickman L., & Rog D. (Eds.), Scale development: Theory and applications (4th ed., pp. 153–204). Los Angeles, CA: Sage Publications.

[nop2508-bib-0005] Ginn, G. O. , Shen, J. J. , & Moseley, C. B. (2009). Community benefit laws, hospital ownership, community orientation activities and health promotion services. Health Care Management Review, 34(2), 109–118. 10.1097/HMR.0b013e31819e90e0 19322042

[nop2508-bib-0006] Green, L. W. , & Kreuter, M. W. (2005). Health promotion planning: An educational and ecological approach. New York, NY: McGraw‐Hill Humanities.

[nop2508-bib-0007] Institute of Medicine Committee for the Study of the Future of Public, H (1988). The future of public health (pp. 411–412). Washington, DC: National Academies Press (US) 10.17226/1091

[nop2508-bib-0008] Kohli, A. K. , & Jaworski, B. J. (1990). Market orientation: The construct, research propositions and managerial implications. Journal of Marketing, 54(2), 1–18. 10.1177/002224299005400201

[nop2508-bib-0009] Kreuter, M. W. (1992). PATCH: Its origin, basic concepts and links to contemporary public health policy. Journal of Health Education, 23(3), 135–139. 10.1080/10556699.1992.10616276

[nop2508-bib-0010] Lin, C. J. , Hsu, C. H. , Li, T. C. , Mathers, N. , & Huang, Y. C. (2010). Measuring professional competency of public health nurses: Development of a scale and psychometric evaluation. Journal of Clinical Nursing, 19(21–22), 3161–3170. 10.1111/j.1365-2702.2009.03149.x 20704628

[nop2508-bib-0011] McClelland, D. C. (1973). Testing for competence rather than for" intelligence.". American Psychologist, 28(1), 1–14. 10.1037/h0034092 4684069

[nop2508-bib-0012] Meyer, J. P. , & Allen, N. J. (2004). TCM employee commitment survey academic users guide. London ON: The University of Western Ontario, Department of Psychology Retrieved from http://employeecommitment.com/TCM‐Employee‐Commitment‐Survey‐Academic‐Package‐2004.pdf

[nop2508-bib-0013] Ministry of Health and Medical Service (2015). National Strategic Plan 2016–2020. Suva, Fiji: Ministry of Health and Medical Service Fiji Retrieved from http://www.health.gov.fj/wp‐content/uploads/2018/03/Strategic‐Plan‐2016‐2020‐Executive‐Version.pdf

[nop2508-bib-0014] Mokkink, L. B. , Prinsen, C. A. C. , Patrick, D. L. , Alonso, J. , Bouter, L. M. , De Vet, H. C. , & Terwee, C. B. (2018). COSMIN methodology for systematic reviews of Patient‐Reported Outcome Measures (PROMs)–user manual. Amsterdam, The Netherlands: Department of Epidemiology and Biostatistics, Amsterdam Public Health research institute Retrieved from https://www.cosmin.nl/wp‐content/uploads/COSMIN‐syst‐review‐for‐PROMs‐manual_version‐1_feb‐2018.pdf

[nop2508-bib-0015] Oshio, S. (2004). Chapter VII. Factor analysis: Reliability and validity In Oshio S. (Ed) Psychologic survey analysis by SPSS & AMOS (pp. 143–158). Tokyo, Japan: Tokyotosho. (Japanese).

[nop2508-bib-0016] Prinsen, C. A. , Mokkink, L. B. , Bouter, L. M. , Alonso, J. , Patrick, D. L. , De Vet, H. C. , & Terwee, C. B. (2018). COSMIN guideline for systematic reviews of patient‐reported outcome measures. Quality of Life Research, 27(5), 1147–1157. 10.1007/s11136-018-1798-3 29435801 PMC5891568

[nop2508-bib-0017] Proenca, E. J. (1998). Community orientation in health services organizations: The concept and its implementation. Health Care Management Review, 23(2), 28–38. 10.1097/00004010-199804000-00004 9595308

[nop2508-bib-0018] Shuster, F. G. (2010). Community assessment and evaluation In Stanhope M., & Lancaster J. (Eds.), Foundation of nursing in the community (p. 218). Louis MO: Mosby.

[nop2508-bib-0019] Spencer, L. Y. S. , & Spencer, S. (1993). Definition of a “Competency” In Spencer L. Y. S., & Spencer S. (Eds.), Competency at work (pp. 9–15). New York, NY: John Wiley & Sons.

[nop2508-bib-0020] Streiner, D. L. , Norman, G. R. , & Cairney, J. (2015). Selecting the items In Streiner D. L., Norman G. R., & Cairney J. (Eds.), Health measurement scales: A practical guide to their development and use. New York, NY: Oxford University Press, USA.

[nop2508-bib-0021] Tanabe, S. , Yanagisawa, S. , Waqa‐Ledua, S. , & Tukana, M. (2019). Identifying characteristic features of community orientation among community health nurses in Fiji. Nursing Open, 6(3), 1113–1123. 10.1002/nop2.305 31367437 PMC6650669

[nop2508-bib-0022] Wiseman, V. , Lagarde, M. , Batura, N. , Lin, S. , Irava, W. , & Roberts, G. (2017). Measuring inequalities in the distribution of the Fiji Health Workforce. International Journal for Equity in Health, 16(1), 115 10.1186/s12939-017-0575-1 28666460 PMC5493125

[nop2508-bib-0023] World Health Organization Regional Office for the Western Pacific (2011). Assessment of health care system In The Fiji Islands Health System Review, Health Systems in Transition 1 (1) (pp. 118–128). Manila, Philippines: WHO Regional Office for the Western Pacific Retrieved from http://iris.wpro.who.int/handle/10665.1/5534

[nop2508-bib-0024] World Health Organization Regional Office for the Western Pacific (2015). The first 20 years of the journey towards the vision of Healthy Islands in the Pacific. Manila, Philippines: WHO Regional Office for the Western Pacific Retrieved from http://www.wpro.who.int/southpacific/publications/healthyisldpacific_web_jas.pdf

